# Functional shortcuts in language co-occurrence networks

**DOI:** 10.1371/journal.pone.0203025

**Published:** 2018-09-11

**Authors:** Woon Peng Goh, Kang-Kwong Luke, Siew Ann Cheong

**Affiliations:** 1 Interdisciplinary Graduate School, Nanyang Technological University, Singapore, Singapore; 2 Complexity Institute, Nanyang Technological University, Singapore, Singapore; 3 School of Humanities, Nanyang Technological University, Singapore, Singapore; 4 School of Physical and Mathematical Sciences, Nanyang Technological University, Singapore, Singapore; University of Southern California, UNITED STATES

## Abstract

Human language contains regular syntactic structures and grammatical patterns that should be detectable in their co-occurence networks. However, most standard complex network measures can hardly differentiate between co-occurence networks built from an empirical corpus and a body of scrambled text. In this work, we employ a motif extraction procedure to show that empirical networks have much greater motif densities. We demonstrate that motifs function as efficient and effective shortcuts in language networks, potentially explaining why we are able to generate and decipher language expressions so rapidly. Finally we suggest a link between motifs and constructions in Construction Grammar as well as speculate on the mechanisms behind the emergence of constructions in the early stages of language acquisition.

## Introduction

The pioneering linguist de Saussure defined language as “a system of interdependent terms in which the value of each term results solely from the simultaneous presence of the others” [1]. Under this definition, it becomes reasonable to employ the framework of complex networks in linguistic studies. The network approach is especially appropriate in analyzing the complex relationships among components in a complex system [2–4]. In the case of language, networks relate words or other linguistic components within the context of semantic, syntactic, co-occurring, or other types of relationships. Semantic networks have been shown to possess small-world and scale-free properties [5, 6]. The syntactic relationship between linguistic components has also been examined by building co-occurring word networks [7, 8] and with networks of syntactic dependencies [9, 10]. Such networks also display the same small-world and scale-free properties.

The works cited above have largely concentrated on the macro-structural properties of language networks. These global approaches, however, are unable to detect grammatical/syntactic structures in the networks. For instance, it has been found that measures like mean path length [11], mean degree [11, 12], and mean clustering coefficient [11, 12] are not significantly different in syntactic networks and non-syntactic networks derived from scrambled text. In fact, Zipf’s Law, the scale-free distribution of word frequencies said to be responsible for the scale-free topology of language networks, can be derived from various language models that do not consider syntax at all [13–15]. An exception is noted in [12] which introduced a selectivity measure that distinguished shuffled text from real ones. Given that the existence of grammatical patterns is universal in all human languages, should these patterns manifest in localized regularities in the language network as detectable micro-structures? If so, then the detection of such micro-structures becomes a generalized process to characterize the grammar of any language since the network approach is universal.

Ultimately, the macro and micro-structures of language exist to serve the purpose of efficient communication. Human languages are vastly more complex than any other forms of animal communication [16–19]. Tellingly, the vastness of the lexicon and the recursive application of structural patterns allow language an almost infinite creative potential in producing meaningful utterances [20, 21]. Despite the complexity and variety of language, the processes of creating and understanding expressions are accomplished remarkably rapidly [7]. This efficiency can be partially attributed to the small-world organization [22] in both semantic and syntactic networks [5, 7, 23–25]. If detectable micro-structures reside within language networks, is it possible that they also play a role accelerating the speed of network access and navigation?

In this work, we adopt an unsupervised learning algorithm developed by Solan *et al*. [26], called the Motif EXtraction (MEX) algorithm that identifies micro-structures called motifs from patterns in word-to-word networks of natural languages and other sequential data. The MEX algorithm has been shown to be capable of learning syntax of both artificial and natural languages. In the first instance, MEX extracted motifs from synthetic context-free grammar corpora that correspond very closely to their underlying production rules [26]. In natural language, the motifs learnt by MEX from the ATIS-2 English language corpus were used to generate novel sentences that were largely judged to be grammatical [26]. Here, we propose and demonstrate that motifs extracted by MEX in language networks are not only objects to represent syntax but also serve as effective and efficient navigational shortcuts in the production and deciphering of language. We also show that motif densities are drastically different between real corpora and their non-syntactic (i.e. scrambled) equivalents, examine how structural properties of motifs evolve through different embedding levels, and speculate on how these motifs arise during language acquisition.

## Complex-network approaches to the study of languages

There is now a large body of work applying complex-network approaches to the study of languages. In this section, we will review how complex-network methods have been used to analyze the styles of different authors [27–29], analyze the multi-layer structure of languages [30–32], identify documents with similar contents [33–35], and in doing scientific research, what references should we consider and which are the most important ones [36].

In literary circles, it is common for an author to use a pen name instead of his or her real name. Some authors even use multiple pen names, depending on which genres, or which age groups of readers they are writing for. On the other hand, we can also have multiple for-hire authors, writing different books of a series under the same pen name. Naturally, we expect the habits of different authors to be different, and these would manifest themselves as different literary styles. To detect different writing styles, Segarra *et al*. constructed the network of *function words* (also called *stop words*) [27]. The links between function words are weighted, to distinguish different separations between the function words, and also to allow these separations to be averaged. Treating a weighted network as a Markov chain, and using the relative entropy as the distance between Markov chains, the weighted networks between different texts can then be compared using hierarchical clustering. If there are not too many authors and styles, but many texts for each style, the different styles (and hence authors) can be accurately identified. In contrast, Amancio *et al*. removed stop words from a text to work only with semantically meaningful words, which they further mapped to their singular and infinitive forms before linking adjacent words to form a word-to-word network. They then combined network features with linguistic features such as word frequency, intermittency, *n*-grams, and used machine learning techniques to identify the authors of various texts with reasonable accuracy [28, 29].

Whether written or spoken, we can break words down into alphabets or phonemes, which are then aggregated into words, and in turn aggregated into phrases, sentences, and higher-level organizations where syntax and semantics emerge. Therefore, we should think of a language as multiple layers of linguistic entities interacting within layers and also between layers. In Ref. [30], Liu and Cong investigated the differences and similarities between the semantic, syntactic, co-occurrence, and phonemes layers of modern Chinese, while by comparing the multilayer structures of Croatian and English [31, 32], Martinčić-Ipšić and her co-workers found universal structural properties regardless of the language at the word-level layers, whereas at the syllabic subword-level, there are more language-dependent structural properties.

Going further, we might also wonder whether network-based approaches can help us get at the meaning behind a text. Traditional ways to do this would be to automatically identify keywords from a text, or to extract the list of *n*-grams from the text, so that to compare multiple texts at the semantic level, all we need to do is to compare the lists of keywords or the lists of *n*-grams. In fact, complex-network approaches can be used to discover the keywords [35]. To disambiguate between words with different meanings, Silva and Amancio combined traditional classifiers like those mentioned above, and pattern-based network classifiers, to demonstrate machine learning accuracies in excess of 70% [33]. Realizing that genre is frequently correlated with style, which manifests itself in the structure of the text, Amancio and co-workers used machine learning techniques to compute the semantic similarity between texts [34]. They found that topological measurements on the network in conjunction with semantic features give the best performance.

Finally, using the semantic comparison methods they have developed, Amancio *et al*. tested the idea of whether it is possible for researchers to do an automatic survey of the literature using a prescribed set of keywords to discover a corpus of related papers. By incorporating citation information for this set of papers, Amancio *et al*. were able to discover the subset of papers which can be considered seminal or highly related [36].

## Syntactic network of languages

There are two general approaches in constructing the syntactic networks of languages. The first is with a syntactic dependency network [9, 10, 23] obtained usually from corpora that have been manually annotated with dependency trees [37]. The second is through means of a co-occurrence network [8, 11] which models the linear ordering of words (or other linguistic units) [38] in a corpus. We focus on the latter technique because the former requires expert guidance in generating the dependency trees whether it is done manually or automatically (algorithms for parsing require supervised training data). Using dependency networks also precludes the analysis of very large or new corpora, esoteric languages with no existing training sets, as well as languages with unknown grammars.

**Fig 1:**
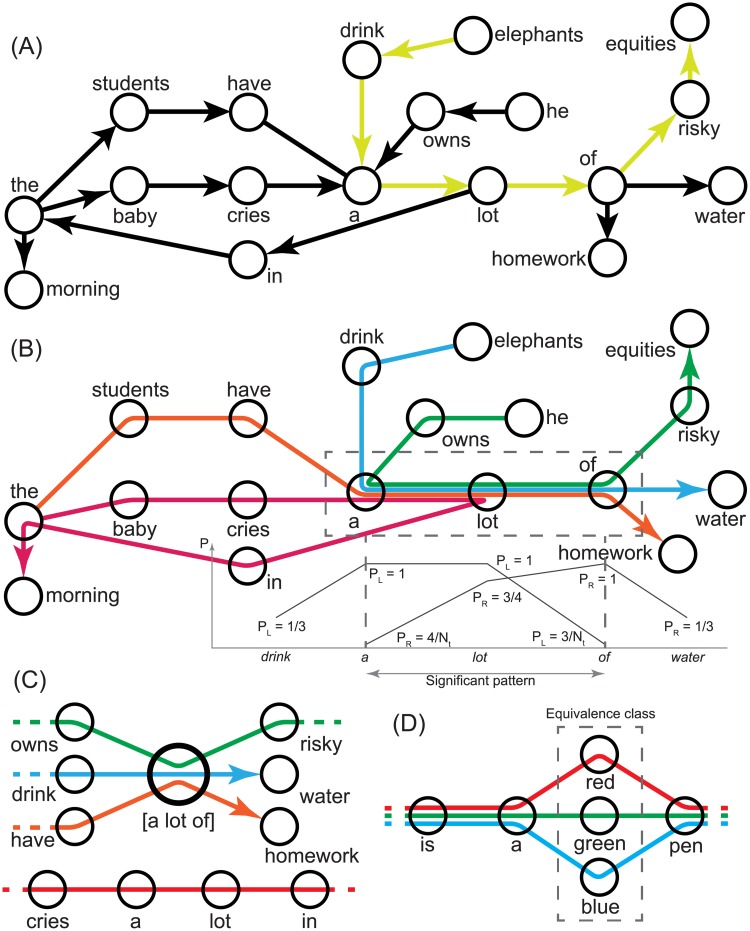
Toy co-occurrence network and pseudograph. (**A**) shows the co-occurrence network based on Sentences 1 to 4. We draw a directed edge between all adjacent word pairs. The unrestricted path from *elephants* to *equities* is highlighted in yellow. The unrestricted distance between the two words is 6. The pseudograph of the same toy corpus is shown in (**B**). Each sentence is represented by a different colored path on the network. A restricted path from *elephants* to *equities* does not exist. The box highlights the motif *a lot of*. It is marked by a fan-in of edges at the start (indicated by the sharp drop in the leftward extension probability *P*_*L*_) and a fan-out at *of* (indicated by the sharp drop of the rightward extension probability *P*_*R*_). A new node for the significant motif is created in (**C**) and the edges are routed through it. (**D**) identifies an equivalence class within a window *L*_*w*_ = 4. An equivalence class ‘supernode’ will be created and the edges are now routed through this new node. However, the unlike motif nodes, the new equivalence class node does not reduce distances between the terminal word nodes that lie on either side of it.

The most straightforward method of constructing co-occurrence networks is to simply draw an edge (directed or undirected) for all pairs of neighbouring words (distance *d* = 1) in a corpus. With this, we risk missing out on interactions that take place at longer distances (i.e. *d* ≥ 2). Fig 1A illustrates a co-occurrence network converted from a small toy corpus of the following 4 sentences:

1. Elephants drink a lot of water.
2. He owns a lot of risky equities.
3. The students have a lot of homework.
4. The baby cries a lot in the morning.

The simplicity of constructing co-occurrence networks means that it can be applied for any language. Even artificial ones can be cast on a network as long as the language is tokenizable. That is, the corpus can be split into tokens of linguistic units and expressed linearly with tokens arranged one after another. However, keeping only short-range correlations while discarding the long-range ones produces an over-generalized network. In the toy corpus, the words *elephants* and *equities* do not appear in the same sentence nor should they do so in normal English usage. Yet, in the network in Fig 1A, one can easily navigate along an unrestricted path *p*_*ur*_ from *elephants* to *equities* and produce a grammatical but unlikely phrase *elephants drink a lot of risky equities*. Clearly, not all possible paths in a co-occurrence network are accessed, or even allowed, in regular language use. In the later sections of this paper, mentions of language/linguistic networks will refer to such co-occurrence networks.

To overcome this over-generalization, Solan *et al*. [26] proposed a pseudograph which limits the number of allowable paths in the network. Instead of having edges that merely join pairs of words, edges in the pseudograph span multiple nodes (i.e. words). Multiple edges between nodes and loops are also allowed. In [26], each edge in the pseudograph connects all the words in a sentence in a linear order. Fig 1B shows the pseudograph of the same toy corpus. Since allowable paths must trace the available edges, a restricted path *p*_*r*_ (not to be confused with the rightward extension probability *P*_*R*_) that leads from *elephants* to *equities* does not exist. Although the pseudograph does not lend itself well to traditional complex network analysis, the same work uses such a network to detect micro-structures called motifs. These motifs appear as coherent bundles of edges spanning short sequences of words and are bookended at both ends with the convergence (fan-in) and divergence (fan-out) of edges. In the example in Fig 1B, the sub-sequence *a lot of* represents such a motif. The procedure of motif detection is described in detail in the methodology section. The motif is then embedded back into the corpus as a single linguistic unit (i.e. a single node, see Fig 1C), acting essentially as a shortcut by reducing network distances between words on opposite sides of the condensed phrase. Here, let us note that the hierarchy of motif embedding is related, but not entirely similar to the multi-layer network representation of language discussed earlier [30–32].

The idea of using network representations to model language goes back a long way (e.g. Jean Aitchison’s book on the mental lexicon [39]). With recent advances in network modelling of human cognition, more and more is known about how word and syntactic structure are stored and accessed in the human mind [40]. Although there is little direct evidence of language networks, experiments have suggested that (i) degrees and local clustering coefficients in phonetic language networks [40] influence speed and accuracy in aural word recognition [41–43]; and that (ii) PageRank (a variant of network eigenvector centrality) of word nodes of the semantic network is better than raw frequencies in predicting human performance in language fluency tasks [44]. Moreover, it is thought that dependency crossings are minimized in the sequential order of sentences to optimize cognitive efficiency [45, 46]. The combined findings all indicate that networks can indeed be useful tools in modelling language processes in the human mind.

It has also been suggested that generating and deciphering language can be usefully modelled as navigation on language networks by means of various strategies [47] such as random walks [48], switching random walks [49], random walks with memory [50], and using ‘landmarks’ with high network closeness centralities [51]. We further propose that linguistic networks contain shortcuts that optimizes their ease of navigation and access. The speed at which humans process language leads us to believe that syntactic networks are not navigated at a purely word-to-word level as this would entail longer path lengths (≈ 20 words, the average length of a sentence) than can be stored in our much smaller working memories of 7 ± 2 objects [52, 53]. Shortcuts would allow us to traverse multiple nodes at a time, shortening the effective path lengths and utilizing less cognitive effort. We will demonstrate in this paper that the motifs detected are not just effective but also efficient network shortcuts.

## Results

### Motif detection in language networks

We applied the Motif EXtraction algorithm (MEX) [26] on three English corpora approximately 10^6^ words in length. They are: i) the Uppsala Student English Corpus (USEC) (1007839 tokens, 22471 words, mean sentence length *μ*_*sent*_ = 19.6) [54] of essays collected from 1999 to 2001 from students at Uppsala University in Sweden taking English as a second language which represents a learner’s corpus; ii) the Brown Corpus (BC) (988331 tokens, 41018 words, *μ*_*sent*_ = 25.3) [55] which is compiled from samples of mainly professionally written American English text complied in the 1960s to represent English usage at a high level of sophistication; and iii) a single-author corpus (SAC) (757542 tokens, 14456 words, *μ*_*sent*_ = 19.2) of Jane Austen’s writings freely available from Project Gutenberg [56] to represent also high proficiency but from a single user’s perspective. The drop threshold parameter is set *η* = 0.65 and the context window length is *L*_*w*_ = 6 following [26] which selected these criteria based on the optimal trade-off between precision and recall.

**Fig 2:**
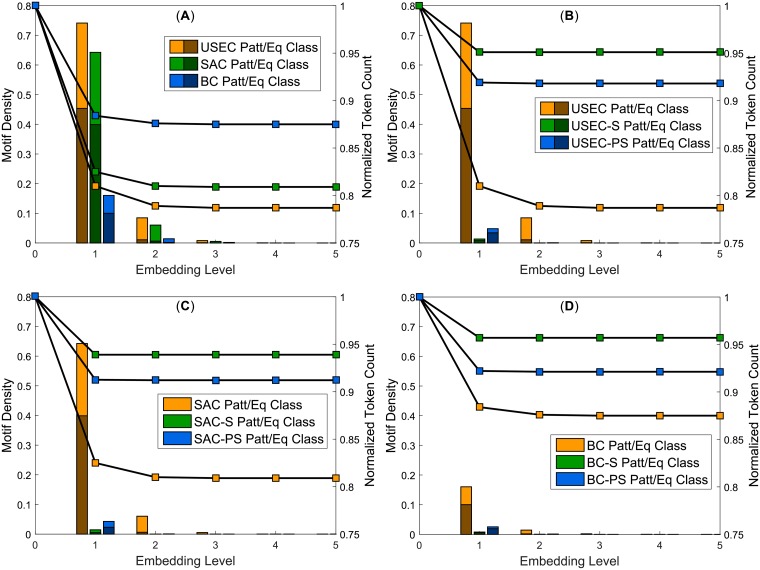
Results of MEX embedding. The bar graphs in the sub-plots show the motif densities (number of motifs divided by the number of original terminal words) and the line plots chart decrease in the number of tokens $N_{toks}^{k}/N_{toks}^{0}$ as more motifs are embedded in the network. (**A**) gives the results for the real corpora (USEC, SAC, and BC) while (**B**), (**C**), and (**D**) show the difference between each real corpus, its shuffled equivalent and its POS-shuffled equivalent.

MEX found the greatest number of patterns/motifs and equivalence classes (collectively termed objects) in the USEC. Fig 2A details the results. For example, at level 1, we detected (6466 + 10191)/22471 = 0.74 as many objects as there were terminal words in the original corpus (i.e. the motif density). It is followed closely by the SAC with 0.64 while the BC lags substantially with 0.16. We define corpus compression by measuring the change in the number of tokens *ΔN*_*toks*_ remaining in the corpus at each embedding level. It is greatest for the USEC which at level 5 contains 794922/1007839 = 0.79 as many tokens as it did at level 0 (i.e. the normalized token count) and has a mean sentence length *μ*_*sent*_ = 15.4. The SAC and the BC follow at 0.81 and 0.88 respectively. With other network measures (see Table 1), the BC also stands out with its very low clustering coefficient *C* [22] and density *ρ* when compared to the other two real corpora (1.1 vs. 3.1 and 4.4, all ×10^−2^).

**Table 1:** Table of network measures. The measurements are density *ρ*, average degree 〈*k*〉, clustering coefficient *C*, assortativity *r*, average minimum unrestricted distances 〈*min*(*d*_*ur*_)〉 (i.e. distances along unrestricted paths as in Fig 1A), average minimum restricted distances 〈*min*(*d*_*r*_)〉, and average mean restricted distances 〈*mean*(*d*_*r*_)〉 (i.e. distances along restricted paths as in Fig 1B). For scrambled (appended with -S) and POS scrambled (appended with -PS) corpora, the values are given up to the precision not affected by fluctuations in the random scrambling.

Corpus	*ρ* × 10^4^	〈*k*〉	*C* × 10^2^	*r*	〈*min*(*d*_*ur*_)〉	〈*min*(*d*_*r*_)〉	〈*mean*(*d*_*r*_)〉
**USEC**	5.1	23.0	3.05	−0.21	3.08	8.97	11.03
**SAC**	9.7	28.1	4.41	−0.26	2.89	16.10	20.70
**BC**	2.3	19.2	1.10	−0.19	3.09	9.79	11.48
**USEC-S**	8.4	37.5	7.66	−0.24	2.96	8.37	10.57
**USEC-PS**	7.8	34.9	5.43	−0.24	2.85	8.71	10.62
**SAC-S**	15.2	44.1	9.34	−0.29	2.83	15.52	20.24
**SAC-PS**	13.8	40.0	7.20	−0.29	2.78	15.73	20.32
**BC-S**	3.2	25.9	2.73	−0.18	3.03	9.44	11.18
**BC-PS**	2.9	23.7	1.61	−0.20	2.98	9.99	11.48

We then tested MEX on artificially-generated corpora with little or no syntactic structures of real language. In Fig 2B, the word order in each sentence of the USEC was shuffled to produce the *shuffled corpus* (USEC-S) and the *POS-shuffled corpus* (USEC-PS) was generated by swapping words of the same Part-of-Speech (POS) category within the corpus. The POS categories for the USEC and SAC were created using a Maximum Entropy tagging algorithm implemented through Python’s Natural Language Toolkit (NLTK) while the BC came with the POS tags included. As expected, only a negligible number of objects were identified in the scrambled corpora. The USEC-S and USEC-PS had motif densities of 0.01 and 0.05 respectively compared to 0.74 of the original USEC at level 1 and at level 2, the ratios of objects extracted from the USEC to its scrambled equivalents are even larger. However, we noted that the scrambled corpora have remarkably higher *C*, *ρ*, and average degree 〈*k*〉 (Table 1). The relatively low *ρ* and 〈*k*〉 in the USEC indicate selectivity in edge formation relative to scrambled corpora. Edges are selective in the sense that some connections in the unscrambled network (e.g. *to* to *the*) are in fact accessed many times in the corpus and after scrambling, these words become adjacent to a larger diversity of neighbours than they originally were.

The MEX algorithm identified motifs from overlaps in the word-to-word pseudograph. The BC is purposefully curated to include a diverse range of genres, subjects, and authorship to represent a wide range of American English usage and thus likely contains only a small number of overlaps in its network structure. Accordingly, the algorithm detected only a small number of motifs in this corpus. The USEC and the SAC, on the contrary, have much greater overlap densities. The USEC is built from students’ essays written on a limited number of topics, with many essays per topic, guaranteeing content overlap. The SAC, composed of only 8 books from Jane Austen, contains both content and stylistic overlaps. It is apparent that selecting a corpus with the appropriate overlap density is more crucial for MEX to detect motifs than it is to choose a large corpus. The network measures *C*, *ρ*, 〈*k*〉, and assortativity *r* [57] proved to be unreliable in determining whether the word-to-word network contains MEX-detectable syntactic regularities. In real corpora, increases in *C*, *ρ*, and 〈*k*〉 seem to correlate loosely with a larger motif density. However, the correlation is completely reversed with the scrambled corpora in the sense that they register hardly any objects in MEX despite possessing higher *C*, *ρ*, and 〈*k*〉. This agrees with the results of [11] which found that 〈*min*(*d*_*ur*_)〉, 〈*k*〉, and *C* are slightly, but not significantly, higher for non-syntactic networks compared to their syntactic equivalents.

### Network distances

Network distances will invariably shrink when a sequence of words is condensed into a single-node shortcut as it does under MEX. We quantify the evolution of network distances under MEX and compare it with an *equal-cost null model* (see methodology section) to demonstrate that MEX motifs are efficient in shrinking network distances. We consider distances only along paths *p*_*r*_ already existing on the pseudograph (i.e. the restricted distances). The reason is two-fold: i) as stated, many of the unrestricted paths are not accessed, or even allowed, in regular language use and ii) as shown in Table 1, unrestricted distances 〈*min*(*d*_*ur*_)〉 are much smaller (≈ 1/3) than the observed restricted distances 〈*min*(*d*_*r*_)〉 in the network. This is effectively what Margan *et al*. have found in Ref. [31].

**Fig 3:**
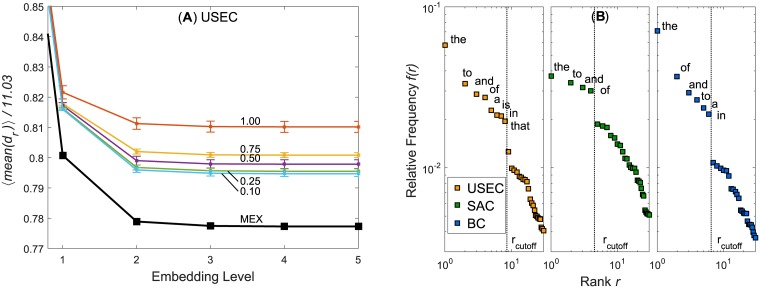
Shrinking distances in the USEC and ranked word frequencies. (**A**) show how 〈*mean*(*d*_*r*_)〉 decreases in the USEC and how it compares to null models set at different cost parameters Γ_*Node*_. (**B**) shows relative the word frequencies *f*(*r*) against rank *r* and highlights the stop word cutoff in each corpus. The cutoffs mark sudden drops in the ranked word frequencies.

The decrease of average mean network distances 〈*mean*(*d*_*r*_)〉 are charted in Fig 3A and S2(A) and S2(B) Fig (see S2(C)–S2(E) Fig in for 〈*min*(*d*_*r*_)〉). At level 5, the maximal layer of embedding, 〈*mean*(*d*_*r*_)〉 for the USEC, SAC, and BC are respectively 8.571/11.027 = 0.777, 16.637/20.696 = 0.804, and 9.936/11.481 = 0.865 of the values of 〈*mean*(*d*_*r*_)〉 at level 0 (i.e. the normalized distances). These fractional reductions seem large, but to convince ourselves that they are significant we need to compare them against the decrease of 〈*mean*(*d*_*r*_)〉 from null models where the shortcuts are random, and thus meaningless. As we will explain the Methods section, for a proper comparison between empirical distances and null-model distances, something must be kept constant. Since it is extremely difficult to keep the numbers of new nodes and new edges constant, we constructed a family of null models where we can keep the *total cost* of adding new nodes and new edges the same as for the empirical network. Regardless of the node-formation cost Γ_*Node*_, the distances of the MEX-embedded networks are always smaller than the null models (see Methods section). This is especially true when Γ_*Node*_ of the null models are set larger than 0.5. Null model distances shrink and approach the MEX results when Γ_*Node*_ < 0.5. For 〈*min*(*d*_*r*_)〉, null models with Γ_*Node*_ < 0.5 sometimes even surpass MEX in distance reduction (e.g. in SAC and BC). It is not trivial to determine which Γ_*Node*_ yields the most realistic null model. In this, one may be guided by empirically calculating the ratio of new nodes to new edges created at each MEX level *k* and set $\Gamma_{Node}N\left(V_{P}^{\left(k\right)}\right)=\Gamma_{Edge}N\left(E_{P}^{\left(k\right)}\right)$. This assumes that equal cost is expanded in node and edge creation. This yields Γ_*Node*_ between 0.82 to 0.97 which is well within the range where MEX shrinks the network more than the null model in both 〈*mean*(*d*_*r*_)〉 and 〈*min*(*d*_*r*_)〉. The choice of Γ_*Node*_/Γ_*Edge*_ > 0.5 can also be motivated from neuropsychology—the creation of new memory that can be associated to previously acquired knowledge (i.e. edges) requires less effort than remembering a new and isolated piece of information (i.e. nodes) [58].

### Taxonomy of MEX patterns

We also attempted to create a taxonomy of MEX-detected patterns. We use the POS tags attached to each token to create *POS templates* of MEX patterns. For example, the phrase *the united states* would have the template *DT (Determiner) JJ (Adjective) NN (Noun)* as would the phrase *a short man*. Significant POS templates are singled out by how their standard scores (z-score) measure against the null ensemble of patterns $V_{P}^{(k)*}$. The most significant POS templates at the first level is *DT (Determiner) NN (Noun) IN (Preposition)* (e.g. *a lot of*) with *z* = 56.75 and frequency *f* = 371. The next is *IN (Preposition) DT (Determiner)* (e.g. *in the*) (see Table 2 for results of the USEC and S1 and S2 Tables for the SAC and the BC). Additionally, the 6466 MEX pattern motifs at level 1 of the USEC can be represented by 1190 POS templates whereas the null model required twice the number of template types (2290). The relatively small set of POS templates used suggests that motifs at this level are in part characterized by a small class of grammatical/syntactic regularities. Significant templates are also detected in *G*^(2)^ such as *[[IN DT] JJ NN]*. There are no significant (*z* > 3) POS templates beyond *G*^(2)^.

**Table 2:** Motif templates of the USEC. We present for levels 1 and 2 the top 3 ranked (in terms of Z-score) POS templates and stop word templates respectively. The Penn Treebank [60] POS tags are used here. Beyond level 2, there were no regularly used templates. For each level, we also gave the number of template types used by the motifs in the corpus and also the number of template types that appeared in randomly extracted motifs.

Rank	Template	Z	F	Example
**Level 1 POS Templates**: 6466 Motifs; 1190 Types; 2290 Null Types				
**1**	[DT NN IN]	56.75	371	[a lot of]
**2**	[IN DT]	41.70	434	[in the]
**3**	[DT VBZ]	34.00	81	[this is]
**Level 2 POS Templates**: 1662 Motifs; 1312 Types; 1442 Null Types				
**1**	[[IN DT] NN [IN DT]]	12.97	6	[[as a] {consequence, result} [of this]]
**2**	[[IN DT] JJ NN]	12.04	22	[[in the] first place]
**3**	[[IN DT] NN NN]	7.43	11	[[in the] right way]
**Level 1 Stop Word Templates**: 4038 Motifs; 83 Types; 98 Null Types				
**1**	_ the _ of _	39.77	227	[the age of]
**2**	_ a _ of _	26.54	110	[a lot of]
**3**	_ the _ to _	19.46	58	[the right to]
**Level 2 Stop Word Templates**: 353 Motifs; 24 Types; 38 Null Types				
**1**	_ of _ to _	7.88	3	[[in the] ages of {15, 16…} to]
**2**	_ of _	3.81	47	[[as a] matter of fact]
**3**	_ to _ to _	3.37	2	[to {quick, young} to]

Classification templates for patterns can also be constructed from *stop words* [59] which in computational linguistics refer to very common closed-class words such as prepositions, conjunctions, and pronouns with little semantic value. While the determination of POS tags demands expert input, *stop word templates* can be constructed from a purely data-driven approach. The list of stop words is not universal and varies among natural language processing tools. We can, however, adopt an operational definition of stop words from empirical data. In Fig 3B, we plotted the ranked relative word frequencies *f*(*r*) of each corpus on log-log plots and observed that there are sudden drops in frequency after *r* = 8, 4, 6 for respectively the USEC, SAC, and BC. This boundary marks a conservative rank cutoff *r*_*cutoff*_ to define an operational list of stop words for a particular corpus. It is entirely possible that stop words (in the closed-class definition sense) exist beyond these cutoffs but are not reflected in the trend of the data and thus cannot be unambiguously identified. For example, stop words like *the* and *to* are common for all three corpora and others like *is* is USEC-specific.

We employ these stop words to construct the *stop word templates*. Stop word templates are defined by replacing the non-stop words in a motif with a blank. For example, the stop word template of *the {reason, explanation} for* is _ *the* _ *for* _ with the inclusion of blanks also at the front and back. Of the 6466 MEX patterns at level 1 of the USEC, 4038 contain at least one stop word in them and can be classified by a template (Table 2). At *G*^(1)^ of the USEC, we find significant templates in _ *the* _ *of* _ (e.g. *the age of*) (*z* = 39.77, *f* = 227) and *_ a _ of _* (e.g. *a lot of*) (*z* = 39.77, *f* = 227). Similar templates are also found in the other two corpora (see S1 and S2 Tables). Beyond *G*^(1)^, the significance and frequency of stop word templates fall drastically with very few registering *z* > 3. This suggests that at *G*^(> 1)^, motifs stop being organized around these operational lists of stop words. We also observe that at the first level *G*^(1)^, stop words appear in MEX-detected motifs 58 to 83% more frequently than by chance (see S3 Table). The trend is inverted at higher levels *G*^(>1)^ as the rates of stop word usage decrease below that of random noise (e.g. at level 2, 16 to 30% below null rate). Although, stop word templates cannot classify patterns as precisely as POS templates (fewer possible template types), they are nevertheless a good unsupervised alternative.

## Discussion

In linguistics, the theories of Construction Grammar (CxG) describe a family of grammar models in which constructions are the basic units of grammar [61–63]. Constructions, in this framework, are *pairings of form and function* [62, 63]. Form refers to the morphological and syntactic features of the construction while function specifies its semantic features [64]. Consider the idiom *kick the bucket* [62]. One cannot derive its meaning by considering its syntax (the ‘X does something to Y’ form) and its component words separately. In CxG, not only idiomatic expressions, but all constructions are said to be form-function pairings. There is also “a continuum from schematic complex constructions to substantive atomic constructions” [65]. Isolated words and complex grammatical arrangements are treated on equal footing in CxG.

One of the processes through which children acquire constructions is entrenchment [66]. Entrenchment is when a person performs a task successfully enough times, the way they perform this task becomes habitual and automatic. Similarly, MEX detect motifs in language pseudographs by searching for repeated sub-sequences. They are reminiscent of constructions in CxG. Take, for example, the generalized pattern *[i* {*feel, think, believe*} *that]* found in the USEC. All three variants of the pattern are identical in their functions of expressing an opinion. Even though it shares an identical form with a pattern like *[i suggest that]*, MEX considers them to be different entities due to their usage in different contexts. Such form-function pairings in MEX motifs are features found also in CxG. Of course, it would be presumptuous to claim that all motifs in MEX are constructions, or that all constructions in the corpus are detectable with MEX. Nevertheless, MEX remains a useful tool to quickly and crudely process a corpus for possible constructions.

Frequently used constructions become entrenched in the mental grammar [64]. These constructions become mental routines directly accessed without invoking higher schemas [64]. This is consistent with our earlier claim that the language network has to contain shortcuts to account for how rapidly we navigate it. In MEX, significant motifs are similarly entrenched in the network as condensed routines. As shown, motifs reduce observed network distances *d*_*r*_ effectively and efficiently. We believe that the creation of such shortcuts in the mental lexicon comes at the expense of using more memory to store the new linguistic units and the new associations that come with it. This cost, modelled in Γ, is offset by the reduction of network distances which we associate with an increase in language processing speed. Earlier, we alluded to the usefulness of modelling language processes in the human mind as network processes. Here, we suggest that these networks are not merely a simple matter of concatenating adjacent words to form strings, but rather contain complex shortcut structures that can be deduced from motif detection.

We can also speculate on the mechanisms that guide the formation of constructions by investigating the properties of MEX motifs. We showed that incidence rates of stop words in level-1 motifs are higher than expected from the frequencies of the stop words themselves. In syntactic networks of natural languages, stop words are hubs owing to their high usage frequency and their flexible combinatorial potential [24]. It has been shown that stop words only become hubs in the syntax network of a child after the *syntactic spurt* [67] stage of language acquisition [23]. If we assume that level-1 motifs correspond to constructions acquired in early stages of language development, this study lends credence to our observation that stop words are important building blocks of low-level motifs. Therefore, depending on the questions we are interested in, we should not always remove stop words from our word co-occurrence network (as is done, for example, in Refs. [28, 29]).

In linguistics, stop words fall under the category of *function words* [68] which can be prepositions, pronouns, auxiliary verbs, conjunctions, articles, or particles. Function words by themselves have little lexical meaning and, instead, serve to mediate and/or to emphasize the interaction between words. We believe that function words arise necessarily out of cognitive limitations. Before they undergo the syntactic spurt, toddlers have small one-word lexicons [67] which can be easily searched through when producing or deciphering utterances. As the lexicons grow, and as the children start to produce 2-to-3 word phrases [67], the numbers of possible expressions explode combinatorially. The same linear search strategy becomes too slow for real-time linguistic interactions. Function words are constrained by the word category preceding them and they in turn constrain the word category following them. For example, a toddler may say ‘put table/bed/box’ to mean ‘put the toy on the table/bed’ or ‘in the box’. With the introduction of a function word like *on*, after saying ‘put on’, it is much more probable to produce *table* or *bed* than *box*. As such, the function word *on* reduces the search space of the possible productions after it.

## Methods

### Motif EXtraction algorithm

The MEX (Motif EXtraction) algorithm was developed by Solan *et al*. [26] to automatically extract patterns and syntax from language corpora. Consider a corpus of *N*_*sents*_ sentences of varying lengths totaling *N*_*toks*_ tokens made up of *N*_*words*_ unique words. It is useful to visualize this corpus as a pseudograph where each word is represented by a node and each sentence is a directed edge or path going through multiple nodes. As described, motifs are coherent sub-sequences of nodes where a number of edges bundle up and are bookended with fan-ins and fan-outs of edges.

We define a path in this graph as (*e*_*i*_; *e*_*j*_) = (*e*_*i*+1_, *e*_*i*+2_,…,*e*_*j*_) where each *e*_*k*_ represents some word node *w*_*l*_. The *extension probabilities* of such a path are

$$P_{R}(e_{i};e_{j})=\frac{\ell (e_{i};e_{j})}{\ell (e_{i};e_{j-1})},j>i$$ (1)

and

$$P_{L}(e_{j};e_{i})=\frac{\ell (e_{i};e_{j})}{\ell (e_{i+1};e_{j})},j>i.$$ (2)

The function *ℓ*(*e*_*i*_; *e*_*j*_) returns the number of edges that traverse the sequence (*e*_*i*_, *e*_*i*+1_,…,*e*_*j*_). Therefore, the equation for *P*_*R*_(*e*_*i*_;*e*_*j*_) describes the probability that a sub-path (*e*_*i*_, *e*_*i*+ 1_,…,*e*_*j*−1_) is found to extend rightward (or forward) to word *e*_*j*_. Similarly, *P*_*L*_(*e*_*j*_;*e*_*i*_) measures the leftward (or backward) extension probability i.e. the probability that a sub-path (*e*_*i*+1_, *e*_*i*+2_,…,*e*_*j*_) is preceded by word *e*_*i*_. *P*_*R*_(*e*_*i*_;*e*_*i*_) = *P*_*L*_(*e*_*i*_;*e*_*i*_) is simply the occurrence probability of *e*_*i*_. As illustrated in Fig 1B, the start and end of a significant motif are marked by sudden drops in their rightward and leftward extension probabilities. There also is precedence of using such extension probabilities to analyze transitions in phonological linguistic elements in [69, 70]. The *drop ratios* are defined as

$$D_{R}(e_{i};e_{j})=\frac{P_{R}(e_{i};e_{j})}{P_{R}(e_{i};e_{j-1})},j>i$$ (3)

and

$$D_{L}(e_{j};e_{i})=\frac{P_{L}(e_{j};e_{i})}{P_{L}(e_{j};e_{i+1})},j>i.$$ (4)

When *D* < *η*, the drop threshold cutoff, we consider the sub-sequence a significant motif. A new pattern node *e*_*patt*_ = [*e*_*i*+1_, *e*_*i*+2_,…,*e*_*j*_] which contains the condensed sequence is created. Paths that previously went through (*e*_*i*+1_, *e*_*i*+ 2_,…,*e*_*j*_) are now routed through the *e*_*patt*_ pattern ‘supernode’ like in Fig 1C. When there are overlapping significant sub-sequences, we prioritize the one with greater significance based on the smallest combined cumulative binomial probability *B*_*comb*_ = *B*_*R*_ + *B*_*L*_ where

$$B_{R}(e_{i};e_{j})=\sum_{x=0}^{\ell (e_{i};e_{j})}Binom\left(\ell (e_{i};e_{j-1}),x,\eta P(e_{i};e_{j-1})\right),j>i$$ (5)

and

$$B_{L}(e_{j};e_{i})=\sum_{x=0}^{\ell (e_{j};e_{i})}Binom\left(\ell (e_{i+1};e_{j}),x,\eta P(e_{i+1};e_{j})\right),j>i$$ (6)

where

Binom(k;n,p)=(nk)pk(1-p)n-k. (7)

Intuitively, low individual probabilities *B*_*R*_ and *B*_*L*_ mean that there are *fewer* extended sub-sequences than expected and thus the drop ratios are more significant. MEX can also accommodate *equivalence classes* of linguistic units that share the same context. Fig 1D shows an example of an equivalence class contained within a context window of length *L*_*w*_ = 4. The slot for the equivalence class can exist at any position of a context window except at the edges. The equivalence class is then merged into an equivalence class ‘supernode’. As seen, the equivalence class node does not shrink distances in the network.

In our implementation of MEX, we consider the network formed from the original corpus of terminal words to be the 0-th level graph with its collection of vertices and edges(*G*^(0)^ = (*V*^(0)^, *E*^(0)^)). From this, we scan the graph first for all possible level 1 equivalence classes (i.e. the generalization step) and embed them to *G*^(0)^, obtaining *G*^(1)−*OnlyEC*^. We then seek for pattern candidates from all possible sub-sequences in *G*^(1)−*OnlyEC*^ and rank the candidates by *B*_*comb*_. The patterns are embedded into the network starting from the most significant and the process is repeated until no more level-1 pattern vertices $V_{P}^{\left(1\right)}$ are found (i.e. the embedding step). This culminates in the level 1 network *G*^(1)^. This ordering of steps enables the equivalence class detection and pattern detection procedures to bootstrap each other to create more equivalence classes and pattern motifs. The algorithm then moves on to construct *G*^(2)^ and so on until no more new objects are identified. This recursive search creates a kind of hierarchy in the embedding of motifs in the network where motifs at a higher embedding layer are compositions of lower-level motifs akin to hierarchies in syntactic structures [71]. For instance, short sequences of words form simple noun-phrase or verb-phrase type motifs which are assembled into complex sentence segments as a higher-level motif.

### Equal-cost null model for network shortcuts

As we perform the MEX procedure on a linguistic network, we create motif ‘supernodes’ that act as distance-reducing bridges (see Fig 1C where, for example, the distance from *drink* to *water* was reduced from 4 to 2 with the creation of the *[a lot of]* shortcut). We believe that there is a ‘cost’ involved in forming such shortcuts. To calculate this cost, recall that when we derive the pseudograph *G*^(*k*)^ from *G*^(*k*−1)^ using MEX, $N(V_{P}^{\left(k\right)})$ number of motifs are found and embedded into the *G*^(*k*)^ pseudograph as shortcut ‘supernodes’. Concurrently, $N(E_{P}^{\left(k\right)})$ edges are also created to link these new nodes to existing ones. We hypothesize that these new network objects are created in the mental lexicon at the cost of

$$\Gamma \left(G^{\left(k\right)},G^{\left(k-1\right)}\right)=\Gamma_{Node}N\left(V_{P}^{\left(k\right)}\right)+\left(1-\Gamma_{Node}\right)N\left(E_{P}^{\left(k\right)}\right).$$ (8)

where Γ_*Node*_ and Γ_*Edge*_ = 1−Γ_*Node*_, both in [0, 1], model the creation costs per node and per edge respectively. Γ(*G*^(*k*)^, *G*^(*k*−1)^) thus gives the total embedding cost when MEX is performed on the *G*^(*k*−1)^ network.

To determine if such MEX-detected shortcuts are in fact *efficient* in shrinking distances, we compare them against a null model where shortcuts are randomly created. We start with *G*^(0)†^ = *G*^(0)^, which is an instance of the null model of *G*^(0)^. To obtain *G*^(1)†^, we keep selecting random sub-sequences in the network to be condensed into shortcuts until Γ(*G*^(*k*)†^, *G*^(*k*−1)†^) = Γ(*G*^(*k*)^, *G*^(*k*−1)^) i.e. the total cost of creating the random shortcuts becomes equal to that of the MEX shortcuts. The subsequent levels *G*^(2)†^, *G*^(3)†^, etc. are similarly derived from *G*^(1)†^, *G*^(2)†^, etc.

Different *G*^(*k*)†^ null models can be obtained by setting different values of Γ_*Node*_. When the ratio Γ_*Node*_/(1−Γ_*Node*_) is high (Γ_*Node*_ ≈ 1 and Γ_*Edge*_ ≈ 0), the null model *G*^(*k*)†^ will contain a similar number of new shortcut nodes to the MEX-processed corpus (i.e. $N\left(V_{P}^{(k)†}\right)\approx N\left(V_{P}^{\left(k\right)}\right)$). In this scenario, *G*^(*k*)†^ has somewhat fewer new edges (i.e. $N\left(E_{P}^{(k)†}\right)<N\left(E_{P}^{\left(k\right)}\right)$) since the MEX criteria implies that each new shortcut sub-sequence is traversed by more than one sentence path (i.e. more than one edge incident on the new node) whereas the randomly created shortcuts are often only used by a single sentence. Conversely, a low Γ_*Node*_/(1 − Γ_*Node*_) ratio results in a *G*^(*k*)†^ model where the total number of edges incident on the randomly created shortcuts is similar to the original ($N\left(E_{P}^{(k)†}\right)\approx N\left(E_{P}^{\left(k\right)}\right)$) and, by the same argument, a greater number of new nodes (i.e. $N\left(V_{P}^{(k)†}\right)>N\left(V_{P}^{\left(k\right)}\right)$).

### Null rate of pattern properties

In examining the properties of the pattern motifs detected by MEX, it is essential to compare these quantities to a null rate i.e. the properties of the ensemble of patterns detected in level *k* if, instead of being detected with the MEX criteria, they were randomly defined. We take *G*^(*k*)−*OnlyEC*^, which is network *G*^(*k*)^ after embedding the equivalence class nodes but *before* patterns are embedded using MEX, and define the ensemble of null model patterns $V_{P}^{(k)*}$ by selecting an equal-length random sub-sequence in this network for every actual motif sub-sequence found by MEX. The null values of the pattern properties are then computed from this ensemble.

## Supporting information

S1 Fig Embedding example. An example sequence taken from the USEC is shown here at different levels of embedding. At *G*^(0)^, only terminal word nodes exist. At *G*^(0)−*OnlyEC*^, some of the terminal word nodes are embedded inside equivalence class supernodes such as *totally* being embedded within {*quite, totally, entirely*}. At *G*^(1)^, the nodes merged into 3 separate level-1 pattern motifs and at *G*^(2)^ these 3 motifs combine to form a level-2 pattern motif.
(PDF)

S2 Fig Decrease of network distances under motif embedding. **A** and **B** shows the decrease of 〈*mean*(*d*_*r*_)〉 in the SAC and BC and how they compare to null models set at different cost parameters. Sub plots (**C**-**E**) chart the decrease of 〈*min*(*d*_*r*_)〉 and their null models for the USEC, SAC, and BC respectively.
(PDF)

S1 Table POS templates of motifs in the SAC. This table is interpreted in the same manner as Table 2.
(PDF)

S2 Table POS templates of motifs in the BC. The BC uses a different list of POS tags which can be obtained from http://www.comp.leeds.ac.uk/ccalas/tagsets/brown.html. This table is interpreted in the same manner as Table 2.
(PDF)

S3 Table Stop word templates of motifs in the SAC. (PDF)

S4 Table Stop word templates of motifs in the BC. (PDF)

S5 Table Table of pattern properties. Here we show the properties of the patterns extracted by MEX for the first 3 levels. 〈*N*_*len*(*P*) = 2_〉 gives the mean occurrence frequency of length-2 patterns and 〈*N*_*len*(*P*)>2_〉 is for patterns with lengths greater than 2. *F*_*SWinP*_, *F*_*CinP*_, and *F*_*PinP*_ are the proportions of objects in the patterns that are stop words, classes, and lower-level patterns respectively. The values are presented with the difference between the observed values and the null values in parentheses together with the error margin. For example, 〈*N*_*len*(*P*) = 2_〉 for the USEC at level 1 is 44.3 and the null model yields 44.3 − 1.3 = 43.0 with and error margin of ±0.3.
(PDF)

S6 Table Table of stop words. Operationally-defined stop words for each of the 3 corpora is given here together with the the part(s) of speech they belong to.
(PDF)
