# Supplementary material for: Functional shortcuts in language co-occurrence networks
Source: PLoS One. 2018 Sep 11;13(9):e0203025. doi: 10.1371/journal.pone.0203025 (PMC6133353; doi:10.1371/journal.pone.0203025)
Supplement: S1 Fig — An example sequence taken from the USEC is shown here at different levels of embedding. At G(0), only terminal word nodes exist. At G(0)−OnlyEC, some of the terminal word nodes are embedded inside equivalence class supernodes such as totally being embedded within {quite, totally, entirely}. At G(1), the nodes merged into 3 separate level-1 pattern motifs and at G(2) these 3 motifs combine to form a level-2 pattern motif. (PDF) [file pone.0203025.s001.pdf]

S1 Fig

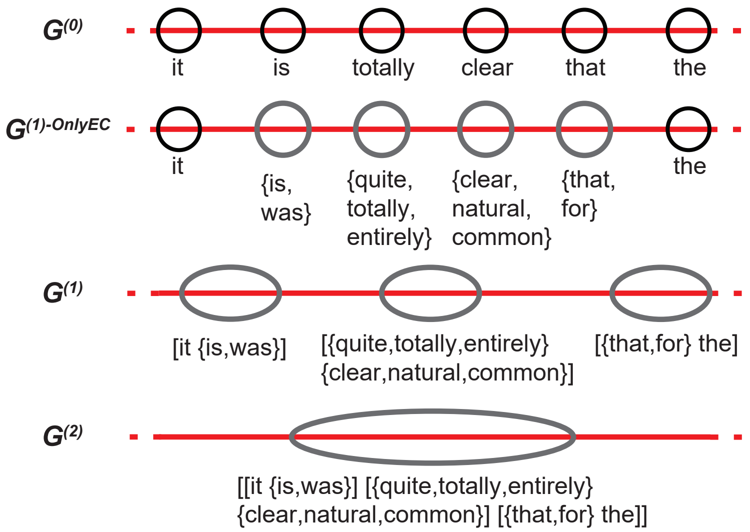

S1 Fig: Embedding example. An example sequence taken from the USEC is shown here at different levels of embedding. At $G^{(0)}$, only terminal word nodes exist. At $G^{(0)-OnlyEC}$, some of the terminal word nodes are embedded inside equivalence class supernodes such as *totally* being embedded within $\{$*quite,totally,entirely*$\}$. At $G^{(1)}$, the nodes merged into 3 separate level-1 pattern motifs and at $G^{(2)}$ these 3 motifs combine to form a level-2 pattern motif.
