# Supplementary material for: Functional shortcuts in language co-occurrence networks
Source: PLoS One. 2018 Sep 11;13(9):e0203025. doi: 10.1371/journal.pone.0203025 (PMC6133353; doi:10.1371/journal.pone.0203025)
Supplement: S2 Fig — A and B shows the decrease of 〈mean(dr)〉 in the SAC and BC and how they compare to null models set at different cost parameters. Sub plots (C-E) chart the decrease of 〈min(dr)〉 and their null models for the USEC, SAC, and BC respectively. (PDF) [file pone.0203025.s002.pdf]

S2 Fig

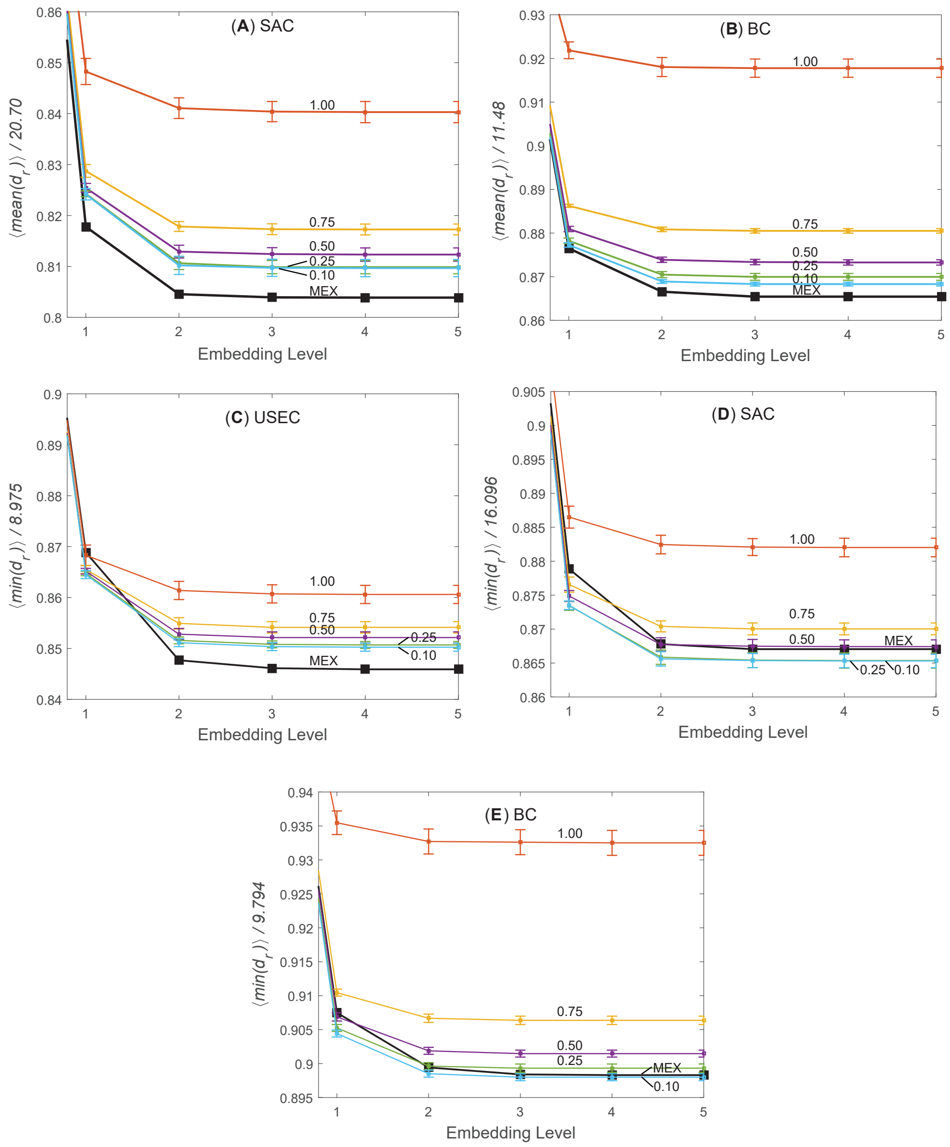

S2 Fig: Decrease of network distances under motif embedding. (**A**) and (**B**) shows the decrease of $\langle mean(d_r) \rangle$ in the SAC and BC and how they compare to null models set at different cost parameters. Sub plots (**C**-**E**) chart the decrease of $\langle min(d_r) \rangle$ and their null models for the USEC, SAC, and BC respectively.
