# Supplementary material for: Functional shortcuts in language co-occurrence networks
Source: PLoS One. 2018 Sep 11;13(9):e0203025. doi: 10.1371/journal.pone.0203025 (PMC6133353; doi:10.1371/journal.pone.0203025)
Supplement: S1 Table — This table is interpreted in the same manner as Table 2. (PDF) [file pone.0203025.s003.pdf]

S1 Table

**Lv**	**Info**		***R***	**Template**	***Z***	***F***	**Example**
**1**	**T**	3522	**1**	[DT NN IN]	57.58	301	[the {exigence,air,...} {for,of}]
	#	647	**2**	[IN RB IN]	27.56	34	[as soon as]
	#*	1392	**3**	[PRP VBD]	25.22	174	[it was]
**2**	**T**	781	**1**	[[IN DT] NN]	11.33	18	[[in the] world]
	#	665	**2**	[[IN DT] JJ NN]	9.17	9	[[in the] first place]
	#*	718	**3**	[[IN DT] NN IN]	6.87	11	[[in the] habit of]

S1 Table: POS templates of motifs in the SAC. This table is interpreted in the same manner as Table 2.
