# Supplementary material for: Functional shortcuts in language co-occurrence networks
Source: PLoS One. 2018 Sep 11;13(9):e0203025. doi: 10.1371/journal.pone.0203025 (PMC6133353; doi:10.1371/journal.pone.0203025)
Supplement: S2 Table — The BC uses a different list of POS tags which can be obtained from http://www.comp.leeds.ac.uk/ccalas/tagsets/brown.html. This table is interpreted in the same manner as Table 2. (PDF) [file pone.0203025.s004.pdf]

S2 Table

Lv	Info		*R*	Template	*Z*	*F*	Example
**1**	**T**	2472	**1**	[AT NN IN]	85.09	319	[a number of]
	#	762	**2**	[PPS BEZ]	31.95	42	[it is]
	#*	1502	**3**	[IN AT]	30.40	158	[of the]
**2**	**T**	513	**1**	[IN NN [IN AT]]	26.39	12	[in spite [of the]]
	#	391	**2**	[[IN AT] NN IN]	26.35	21	[[on the] basis of]
	#*	481	**3**	[[IN AT] NN [IN AT]]	12.02	8	[[at the] end [of the]]

S2 Table: POS templates of motifs in the BC. The BC uses a different list of POS tags which can be obtained from `http://www.comp.leeds.ac.uk/ccalas/tagsets/brown.html`. This table is interpreted in the same manner as Table 2.
