# Supplementary material for: Functional shortcuts in language co-occurrence networks
Source: PLoS One. 2018 Sep 11;13(9):e0203025. doi: 10.1371/journal.pone.0203025 (PMC6133353; doi:10.1371/journal.pone.0203025)
Supplement: S3 Table — (PDF) [file pone.0203025.s005.pdf]

S3 Table

**Lv**	**Info**		***R***	**Template**	***Z***	***F***	**Example**
**1**	**T**	1334	**1**	_ the _ of _	72.27	294	[the sight of]
	#	20	**2**	_ to _ to _	9.59	15	[to speak to]
	#*	23	**3**	_ to _ of _	9.27	11	[to think of]
**2**	**T**	122	**1**	_ of _	3.21	36	[[with a] look of]
	#	5	**2**	_ and _ to _	0.86	1	-
	#*	11	**3**	_ the _	0.49	21	-

S3 Table: Stop word templates of motifs in the SAC.
