# Supplementary material for: Functional shortcuts in language co-occurrence networks
Source: PLoS One. 2018 Sep 11;13(9):e0203025. doi: 10.1371/journal.pone.0203025 (PMC6133353; doi:10.1371/journal.pone.0203025)
Supplement: S4 Table — (PDF) [file pone.0203025.s006.pdf]

S4 Table

Lv	Info		R	Template	Z	F	Example
**1**	**T**	1625	**1**	_ the _ of _	66.66	307	[the number of]
	#	44	**2**	_ a _ of _	33.17	75	[a number of]
	#*	44	**3**	_ in _ of _	16.92	14	[in terms of]
**2**	**T**	154	**1**	_ of _	2.86	38	-
	#	12	**2**	_ to _ the _	2.59	4	-
	#*	15	**3**	_ in _ of _	2.05	1	-

S4 Table: Stop word templates of motifs in the BC.
