# Supplementary material for: Functional shortcuts in language co-occurrence networks
Source: PLoS One. 2018 Sep 11;13(9):e0203025. doi: 10.1371/journal.pone.0203025 (PMC6133353; doi:10.1371/journal.pone.0203025)
Supplement: S5 Table — Here we show the properties of the patterns extracted by MEX for the first 3 levels. 〈Nlen(P) = 2〉 gives the mean occurrence frequency of length-2 patterns and 〈Nlen(P)>2〉 is for patterns with lengths greater than 2. FSWinP, FCinP, and FPinP are the proportions of objects in the patterns that are stop words, classes, and lower-level patterns respectively. The values are presented with the difference between the observed values and the null values in parentheses together with the error margin. For example, 〈Nlen(P) = 2〉 for the USEC at level 1 is 44.3 and the null model yields 44.3 − 1.3 = 43.0 with and error margin of ±0.3. (PDF) [file pone.0203025.s007.pdf]

S5 Table

Lv	Cr	$\langle N_{len(P)=2} \rangle$	$\langle N_{len(P)>2} \rangle$	$F_{SWinP} \times 10$	$F_{CinP} \times 10$	$F_{PinP} \times 10$
1	**USEC**	44.3 (1.3±0.3)	9.4 (6.3±0.1)	2.9 (1.1±0.0)	2.5 (1.9±0.0)	-
	**SAC**	60.0 (10.0±1.0)	8.3 (5.5±0.3)	1.9 (0.7±0.0)	2.4 (2.1±0.0)	-
	**BC**	98.5 (35.2±2.4)	7.4 (5.3±0.3)	3.3 (1.5±0.1)	1.2 (1.1±0.0)	-
2	**USEC**	22.8 (14.0±0.3)	4.9 (3.6±0.1)	0.7 (-0.3±0.1)	2.3 (1.9±0.0)	4.2 (1.9±0.1)
	**SAC**	27.9 (19.0±1.7)	4.8 (3.6±0.0)	0.5 (-0.2±0.0)	2.5 (2.3±0.0)	4.3 (2.2±0.1)
	**BC**	37.5 (29.0±1.7)	5.3 (4.2±0.1)	1.0 (-0.2±0.1)	1.9 (1.8±0.0)	3.9 (2.5±0.1)
3	**USEC**	9.3 (3.8±1.0)	4.3 (3.2±0.0)	0.6 (-0.5±0.1)	2.0 (1.7±0.0)	5.2 (2.9±0.2)
	**SAC**	8.5 (2.2±1.5)	3.7 (2.5±0.1)	0.5 (-0.2±0.1)	3.4 (3.2±0.1)	4.7 (3.0±0.2)
	**BC**	17.5 (10.7±1.3)	3.7 (2.5±0.1)	1.0 (-0.1±0.1)	2.6 (2.4±0.1)	4.7 (3.4±0.2)

S5 Table: Table of pattern properties. Here we show the properties of the patterns extracted by MEX for the first 3 levels. The corpora are stated in the 'Cr' column. $\langle N_{len(P)=2} \rangle$ gives the mean occurrence frequency of length-2 patterns and $\langle N_{len(P)>2} \rangle$ is for patterns with lengths greater than 2. F_{SWinP}, F_{CinP}, and F_{PinP} are the proportions of objects in the patterns that are stop words, classes, and lower-level patterns respectively. The values are presented with the difference between the observed values and the null values in parentheses together with the error margin. For example, $\langle N_{len(P)=2} \rangle$ for the USEC at level 1 is 44.3 and the null model yields $44.3 - 1.3 = 43.0$ with and error margin of ± 0.3.
