# Supplementary material for: Functional shortcuts in language co-occurrence networks
Source: PLoS One. 2018 Sep 11;13(9):e0203025. doi: 10.1371/journal.pone.0203025 (PMC6133353; doi:10.1371/journal.pone.0203025)
Supplement: S6 Table — Operationally-defined stop words for each of the 3 corpora is given here together with the the part(s) of speech they belong to. (PDF) [file pone.0203025.s008.pdf]

S6 Table

USEC		SAC		BC	
**Word**	**Category**	**Word**	**Category**	**Word**	**Category**
**the**	article	**the**	article	**the**	article
**to**	preposition /adverb	**to**	preposition /adverb	**of**	preposition
**and**	conjuction	**and**	conjuction	**and**	conjuction
**of**	preposition	**of**	preposition	**to**	preposition /adverb
**a**	article			**a**	article
**is**	verb			**in**	preposition
**in**	preposition				
**that**	article /conjunction				

S6 Table: Table of stop words. Operationally-defined stop words for each of the 3 corpora is given here together with the the part(s) of speech they belong to.
